# Intraoperative Hemodynamics of Parasylvian Cortical Arteries for Predicting Postoperative Symptomatic Cerebral Hyperperfusion after Direct Revascularization in Patients with Moyamoya Disease: A Preliminary Study

**DOI:** 10.3390/jcm12113855

**Published:** 2023-06-05

**Authors:** Zhiyong Shi, Lingyun Wu, Yi Wang, Wei Li, Juan Wang, Yongbo Yang, Chunhua Hang

**Affiliations:** Department of Neurosurgery, Nanjing Drum Tower Hospital, Nanjing Medical University, Nanjing 210008, China

**Keywords:** moyamoya disease, cerebral hyperperfusion syndrome, parasylvian cortical arteries (PSCAs), risk factors, microvascular doppler ultrasonography, hemodynamics

## Abstract

**Objective**. The search for methods by which to predict the risks of cerebral hyperperfusion syn-drome (CHS) in adults with moyamoya disease (MMD), including those utilizing new biomarkers, still deserves further research. The objective of this study was to investigate the association between the hemodynamics of parasylvian cortical arteries (PSCAs) and postoperative CHS. **Methods**. A consecutive number of adults with MMD who had undergone direct bypass between September 2020 and December 2022 were recruited. Intraoperative microvascular doppler ultrasonography (MDU) was performed to evaluate the hemodynamics of PSCAs. The intraoperative flow direction, mean value of velocity (MVV) of recipient artery (RA) and bypass graft were recorded. According to flow direction after bypass, RA was divided into entering sylvian (RA.ES) and leaving sylvian (RA.LS) subtypes. Univariate, multivariate, and ROC analyses of the risk factors for postoperative CHS were performed. **Results**. A total of 16 (15.09%) cases in 106 consecutive hemispheres (101 patients) sat-isfied the postoperative CHS criteria. According to univariate analysis, advanced Suzuki stage, MVV of RA before bypass, and fold increase of MVV in RA.ES after bypass were significantly associated with postoperative CHS (*p* < 0.05). Multivariate analysis indicated that left-operated hemisphere (OR (95%CI), 4.58 (1.05–19.97), *p* = 0.043), advanced Suzuki stage (OR (95%CI), 5.47 (1.99–15.05), *p* = 0.017), and fold increase of MVV in RA.ES (OR (95%CI), 1.17 (1.06–1.30), *p* = 0.003) were statistically significantly associated with the occurrence of CHS. The cut-off value of fold increase of MVV in RA.ES was 2.7-fold (*p* < 0.05). **Conclusions**. Left-operated hemisphere, advanced Suzuki stage, and postoperative fold increase of MVV in RA.ES were potential risk factors for postoperative CHS. Intraoperative MDU was useful for evaluating hemodynamics and predicting CHS.

## 1. Introduction

Moyamoya disease (MMD) is a relatively rare cerebrovascular disease, characterized by progressive bilateral stenosis of the bilateral terminal portion of the internal carotid artery (ICA) and by the formation of moyamoya vessels at the base of the brain [1]. Extracranial–intracranial (EC–IC) bypass, such as superficial temporal artery–middle cerebral artery (STA–MCA) anastomosis, is considered to be an effective treatment for MMD, one which can reduce the incidence of stroke recurrence and improve long-term survival status [2,3,4]. However, direct bypass revascularization is viewed as a “flow competition” between the EC–IC circulation, causing cerebral hyperperfusion syndrome (CHS) as a common postoperative complication, one which is associated with transient neurological deficits (TNDs) or even intracranial hemorrhage [5]. Consequently, the mechanism of postoperative CHS after direct bypass and its corresponding solutions have important practical value in clinical practice. According to previous reports, left hemisphere, hemorrhagic onset, brain surface temperature, and neurological status on admission were potential risk factors for symptomatic CHS [6,7,8,9].

However, there are few reports concerning the relationship between hemodynamic sources of para-sylvian cortical arteries (PSCAs) and postoperative CHS. Zhang et al. have reported that the hemodynamic source around anastomosis was associated with postoperative CHS, with PSCAs leaving sylvian and anterograde hemodynamic sources from the MCA both being seen as high-risk factors [10]. However, this research was qualitative, not quantitative, and lacked intraoperative hemodynamic details, thus, the mechanisms and hemodynamic predictors of postoperative CHS remained to be fully understood. Moreover, previous literature has confirmed that intraoperative transit-time ultrasonography combined with FLOW800 could predict the postoperative CHS [11]. In this study, we reviewed the intraoperative sources of direction and blood flow in the PSCAs, which are commonly selected as the recipient arteries (RAs). The objective of this study was to investigate the association between the intraoperative hemodynamics of PSCAs and postoperative symptomatic CHS.

## 2. Methods

### Patient Selection

The study was approved by Nanjing Drum Tower Hospital, the affiliated hospital of Nanjing Medical University, and patient consent was obtained. The inclusion criteria of the study were as follows: (1) patients who satisfied the guideline of MMD proposed by the Research Committee on Spontaneous Occlusion of the Circle of Willis of the Ministry of Health, Labor, and Welfare, Japan [12]; (2) patients with a clinical diagnosis of MMD, and treated by STA–MCA anastomosis; (3) intraoperative microvascular doppler ultrasonography (MDU) and FLOW 800 indocyanine green (ICG) color-coded maps were performed to evaluate hemodynamics; (4) patients underwent at least 2 CT perfusion (CTP) scans before and 1 week after surgery to evaluate hemispheric perfusion; (5) a postoperative MRI angiography (MRA) scan was performed to confirm the anastomotic patency; and (6) patients were aged over 18 years. All patients that did not meet these criteria were excluded from this study. A total of 106 hemispheres, operated by 2 surgeons (Y.B.Y. or C.H.H.) in our hospital between September 2020 and December 2022, were recruited for this research.

## 3. Data Collection and Analysis

### 3.1. Hemispheric Perfusion Analysis

The stage of operative hemispheric perfusion was evaluated based on CTP, using the cerebellar cortex as reference [13]. CTP parameters included regional cerebral blood volume (rCBV), regional cerebral blood flow (rCBF), mean transit time (MTT), and time to peak (TTP). The criteria for pre-infarction period were as follows: Stage Ia was delayed TTP, whereas MTT, rCBF, and rCBV were normal. Stage Ib was delayed TTP and MTT, and rCBF and rCBV were normal or slightly increased. Stage IIa was delayed TTP and MTT, decreased rCBF, but normal or slightly decreased rCBV. Stage IIb was delayed TTP and MTT and decreased rCBF and rCBV.

### 3.2. Angiographic Analysis

Angiographic classification was undertaken according to the classic Suzuki stage, with a modified Suzuki stage used in this research. The modified Suzuki stage of the hemisphere was divided into three subgroups as follows: Suzuki stages 1–2 were defined as the early stage, Suzuki stages 3–4 were defined as the middle stage, and Suzuki stages 5–6 were defined as the advanced stage.

### 3.3. Data Collection and Analysis of MDU

The MDU studies were performed with a standard MDU (MultiDop X 16M, DWL, Singen, Germany) with a handheld transducer in a pulsed-wave mode in a single laboratory. All examiners followed the same protocol. All surgical procedures were performed under general anesthesia. The craniotomy was exposed to ensure simultaneous exposure of the PSCAs in the frontal, temporal, and parietal lobes. Thereafter, the operator placed an MDU coupler on each PSCA at an angle of 30 degrees within the craniotomy. During MDU measurement, the cut flow of the PSCA was measured and recorded as mean velocity value (MVV). The PSCA harboring the highest MVV was always selected as the recipient artery (RA). Moreover, the recorded flow direction and MVV of RA was also recorded before anastomosis.

**Figure 1:**
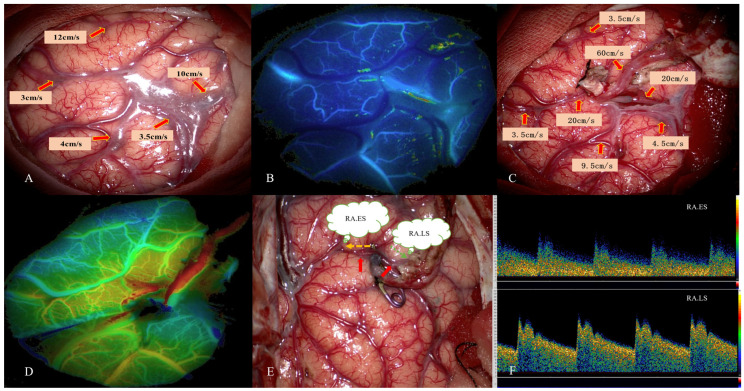
This 45-year-old female patient was diagnosed with MMD due to persistent headache. STA–MCA bypass was performed. Intraoperative images of MDU hemodynamic analysis in PSCAs were obtained. (**A**) Hemodynamics of PSCAs before anastomosis. RA was selected from PSCAs with MVV of 10 cm/s. (**B**) Preoperative blood distribution by FLOW 800 image. (**C**) MDU hemodynamics of PSCAs after bypass. (**D**) Postoperative hemodynamics evaluated by FLOW 800 image. (**E**) Flow direction of RA (red arrow) after bypass. The orange dotted arrow represents RA.ES, and the green represents RA.LS. (**F**) MDU spectrum of RA.ES_post_ and RA.LS_post_ after bypass. This patient had no postoperative CHS. MDU = microvascular doppler ultrasonography, PSCAs = para-sylvian cortical arteries. CHS = cerebral hyperperfusion syndrome. RA.ES = recipient artery entering sylvian. RA.LS = recipient artery leaving sylvian.

Typically, patients undergo STA–MCA bypass involving the anastomosis site of the M4 segment of the ipsilateral hemisphere. According to the flow direction of the MDU measurement after anastomosis, RA was divided into RA entering sylvian (RA.ES) and RA leaving sylvian (RA.LS) subtypes (Figure 1). In addition, the MVV fold change of RA was calculated and defined as RA.ES_post_/RA_pre_ and RA.LS_post_/RA_pre_. Moreover, the flow direction and MVV of the bypass grafting were recorded. Hemodynamics were recorded and summarized, including flow direction and MVV (RA_pre_, RA.ES_post_, RA.LS_post_, and fold change). The MDU studies were conducted and interpreted by 2 experienced neurosurgeons. In most cases, the time needed for surgical anastomosis was less than 20 min, and the patency was subsequently confirmed by intraoperative Flow 800 ICG examination. After that, the dura mater was turned to cover the brain surface, with systolic pressure (cuff pressure) maintained at 110~120 mmHg.

### 3.4. Definition of Symptomatic CHS

For postoperative cases in which there were complaints of discomfort after surgery, emergent MRA and CTP scans were performed to determine the cause. CHS was mainly diagnosed with the following: (1) clinical discomfort, including ipsilateral headache, contralateral muscle strength decrease, contralateral paresthesia, seizure attack, verbal dysfunction, and aphasia; (2) the presence of significant regional CBF increase around the anastomosis site (qualitative observation of an intense focal increase in pre-infarction perfusion stage); (3) apparent visualization of STA–MCA bypass on CTA; and (4) postoperative MRI, including diffusion-weighted imaging (DWI), was performed to exclude possible ischemic pathology. All postoperative radiological results were reviewed by 2 independent neurosurgeons who were blinded to the postoperative evaluation.

## 4. Statistical Analysis

All statistical analysis was performed with IBM SPSS 22.0 (IBM Corp., Armonk, NY, USA). Univariate statistical analysis was performed to assess the association of clinical, radiological, and intraoperative hemodynamics with postoperative CHS. Pearson’s chi-square test was used for categorical variables. An independent *t* test was used for continuous variables with normal distribution, and rank test was used for skew distribution. Multivariate analysis: For the variables with significant statistical differences in univariate analysis (*p* < 0.1), and for the factors that might have a significant influence on symptomatic CHS, binary logistic regression with an enter model was applied to multivariate analysis. The *p* value, the ratio of odds (OR) and the 95% confidence interval (CI) of the ratio were calculated. ROC analysis was performed to establish the cut-off value. A *p* value of <0.05 was considered statistically significant. Finally, Pearson correlation analysis was performed to evaluate the potential hemodynamic risk factor of CHS and other variables.

## 5. Results

### 5.1. Patient Characteristics

A total of 101 consecutive adult patients (106 hemispheres; patient age range 18–64 years, mean 50 years) with MMD satisfied the inclusion criteria in the study; 86 (81.13%) patients had ischemic onset, and 20 (18.87%) patients had hemorrhagic onset. Fifty women (47.17%) and 56 men (52.83%) were enrolled. In 52 of the cases, patients underwent operation on the left hemisphere (49.05%). Patients with past infarction, hypertension, and diabetes comprised 61, 52, and 18 of the cases, respectively. Ipsilateral hemisphere with a Suzuki stage ranging from I to VI comprised 13, 26, 39, 16, 8, and 4 of cases, respectively. The hemispheres with no, sparse and dense moyamoya vessels comprised 19, 35, and 52 of the cases, respectively (Table 1).

### 5.2. MDU Findings in Cases with CHS

**Figure 2:**
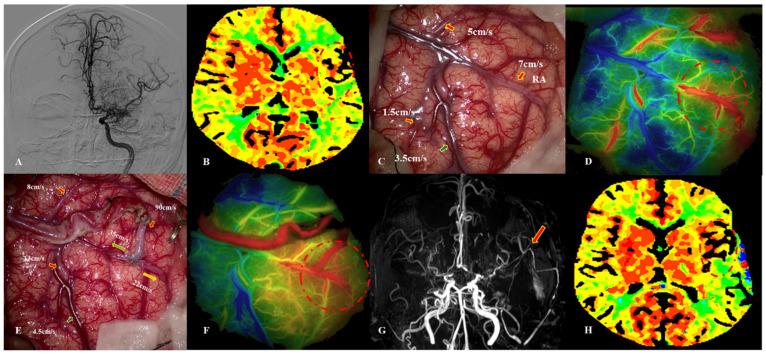
This 39-year-old female patient was diagnosed with MMD due to paresthesia. Intraoperative image of MDU hemodynamic analysis in PSCAs. (**A**) DSA image before surgery. (**B**) CTP showed that TTP was delayed (dotted red circle). (**C**) Hemodynamics of PSCAs before anastomosis (artery: red arrow, vein: blue arrow). RA was chosen from PSCAs with MVV of 7 cm/s. (**D**) Blood distribution by FLOW 800 image before bypass (dotted red circle). (**E**) MDU hemodynamics of PSCAs after bypass, with a 5-fold increase for RA.ES (green arrow) and a 3-fold increase for RA.LS (orange arrow). (**F**) Postoperative hemodynamics evaluated by FLOW 800 image, indicating that regional perfusion improved (dotted red circle). (**G**) MRA demonstrated patency of donor artery (red arrow). (**H**) Postoperative CTP images showed that regional TTP decreased. This patient presented with severe headache and verbal dysfunction at 3 days after bypass. MDU = microvascular doppler ultrasonography, PSCAs = para-sylvian cortical arteries, CHS = cerebral hyperperfusion syndrome, RA.ES = recipient artery entering sylvian, RA.LS = recipient artery leaving sylvian.

Of 16 (15.09%) of 106 hemispheres, postoperative CHS occurred (Table 2). We noted 6 cases of verbal dysfunction, 5 cases of aphasia, 3 cases of cerebral hemorrhage, and 4 cases of severe ipsilateral headache, respectively. Illustrated case was shown in Figure 2. Onset of CHS ranged from 15 min to 5 days post-operation. Out of 16 cases with postoperative CHS, 7 cases of RA.ES and 9 cases of RA.LS were observed. The MVV of RA in cases with CHS were 7.12 cm/s, which differed significantly from those without CHS (*p* < 0.05).

### 5.3. Potential Risk Factors of CHS

**Figure 3:**
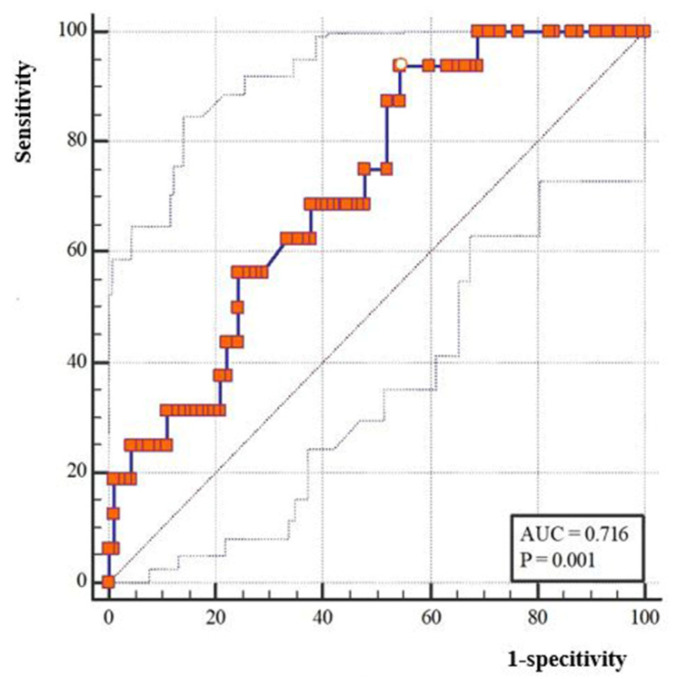
ROC analysis and cut-off value of the MMV fold change of the recipient artery entering sylvian after bypass.

According to univariate analysis, advanced Suzuki stage, MVV of RA before bypass, and fold increase of MVV in RA.ES after bypass were significantly associated with postoperative CHS (*p* < 0.05). Multivariate analysis indicated that left-operated hemisphere (OR (95%CI), 4.58 (1.05–19.97), *p* = 0.043), advanced Suzuki stage (OR (95%CI), 5.47 (1.99–15.05), *p* = 0.017), and MVV fold increase of RA.ES (OR (95%CI), 1.17(1.06–1.30), *p* = 0.003) were statistically significantly associated with occurrence of CHS (Table 3). According to ROC analysis, the cut-off value of the fold increase of MVV in RA.ES was 2.7-fold (*p* < 0.05). The AUC for MVV fold change in RA.ES was 0.716 (Figure 3).

### 5.4. Correlation Analysis between Hemodynamics of RA.ES and Other Variables

After correlation analysis, MVV fold increase was positively correlated with diabetes (r = 0.308, *p* = 0.001) and infarction history (r = 0.252, *p* = 0.009), but negatively correlated with the onset of symptoms (r = −0.222, *p* = 0.022) (Table 4).

## 6. Discussion

Based on the MDU assessment in this study, we can say that the PSCAs that are commonly selected as RA for direct anastomosis in MMD patients receive their blood mainly from entering or leaving the sylvian. The search for predictive risks of CHS, including new biomarkers, still deserves further research.

Postoperative CHS was caused by the contradiction between the impaired cerebrovascular autoregulation of intracranial arteries, increased vascular permeability, and the sudden increase of blood input from the bypass graft [14,15,16]. Previous literature has reported that left-operated hemisphere, hemorrhagic onset, high brain surface temperature, and poor neurological status on admission were risk factors for symptomatic CHS [6,7,8,9]. In this series, we have reported that left-sided operation (OR (95%CI), 4.58 (1.05–19.97)) had a significant association with postoperative CHS, which is consistent with previous reports [7]. We speculate that the major center for language is in the left hemisphere of the brain for most people. Accordingly, verbal dysfunction or even aphasia may be easier to detect when compared with the right where it may be more symptomless. We agree that confounding factors, such as the Suzuki stage or hemispheric perfusion status, might cause result bias when compared with hemispheric difference. Thus, hierarchical chi-square, propensity matched score, or case-control study is a good option for further research.

Moreover, Previous reports have detailed that an advanced Suzuki stage characterized by low flow velocity in MCA is associated with bypass-related infarction [17]. In this study, advanced Suzuki stage had a significant association with postoperative CHS (OR (95%CI), 5.47 (1.99–15.05)). Thus, we hypothesize that MMD cases with an advanced Suzuki stage harbor a stable blood supply pattern from the posterior and extracranial circulation, and that direct bypass might temporarily break this fragile blood perfusion equilibrium, resulting in disturbance of focal hemodynamics. Thereby, we speculatedthat cases with advanced Suzuki stage are prone to receive indirect bypass when there are neurological-related complications; a phenomenon which requires further research.

Thus far, the hemodynamics of PSCAs after bypass are not fully understood. Zhang et al. [10] reported that the blood direction of recipient PSCAs is related to the occurrence of postoperative CHS, which is inconsistent with our results. In this study, the direction of RA was not associated with postoperative CHS (*p* > 0.05), while the MVV of RA before surgery might have been. Univariate analysis reported that the MVV of RA had a significant association with postoperative CHS (*p* = 0.007), whereas multivariate analysis had no significant association (*p* = 0.064). Even if statistical significance was not achieved, we still hypothesize that RA selection from PSCAs with low MVV should be cautious. In other words, direct bypass usually tends towards cases with the highest MVV of RA in clinical practice and might thus be productive in redistributing flow after bypass. Investigation of the deep layer vascular network of the brain might be beneficial in cases where super-selective arterial spin labeling (ASL) perfusion MRI might be an option [18].

In addition to its association with preoperative indicators, CHS is also correlated with postoperative hemodynamics. Wang et al. have reported that CHS is caused by multiple factors, with perfusion change as a risk factor and perfusion range as a protective factor [11]. In this research, the fold change of MVV in RA.ES was a potential risk factor for the occurrence of postoperative CHS. Previous reports have revealed that CHS is an unstable state of cerebral hemodynamics, causing compartmentalization of the cortex as it is perfused by each artery, and a coexistence of local hyperperfusion and hemispheric hypoperfusion [19]. We hypothesize that direct bypass contributes to watershed shift and the new and immediate formation of flow equilibrium. The flow of RA changed from unidirectional to bidirectional after surgery, meaning that it entered and left the sylvian subtypes that harbored different compartmentalized perfusion areas. The flow of RA.ES led to flow redistribution in an adjacent cortex around anastomosis, which we suspect achieved a counterbalance to the cerebral deep vascular network. During this process, deep vascular anastomosis acted as a resistant in balancing blood redistribution. The more the flow of RA.ES, the more redistribution in the adjacent area. In this series, the cut-off value of the MVV change of RA.ES after bypass was 2.7-fold, for which we suspect aggressive blood pressure control and strict fluid input strategy are necessary. Moreover, other reports also support the potential significance of the deep vascular network [20]. Flow pattern in cerebral hemodynamics caused by bypass graft could lead to a watershed shift at the adjacent or remote cortex, which is associated with postoperative cerebral hypoperfusion [5]. We suspect that the cerebral deep vascular network’s role contributed to the formation of the coexistence of hyperperfusion and hypoperfusion after bypass, indicating an unstable and volatile state of hemodynamics. Although the etiology of postoperative CHS remains unknown, our results indicate that the quality of the recipient PSCAs and their deep vascular characteristics might act as critical roles in the occurrence of postoperative CHS. Comprehensive evaluation of ipsilateral Suzuki stage and the intraoperative hemodynamics of recipient PSCAs could determine the increase in regional cerebral blood flow (rCBF) and predict this hyperperfusion complication. Further investigations are needed to properly distribute blood flow from the donor artery toward other regions.

Previous literature has reported that color doppler ultrasonography could be used as a non-invasive imaging modality to quantitatively assess vascular hemodynamics. Wang et al. have confirmed that ultrasonography could predict a degree of collateral neovascularization before and after different kinds of revascularization surgeries [21]. Yeh et al. have reported that collateral development is associated with age after indirect bypass, with a greater increase for pediatric patients than for adults [22]. Zheng et al. have reported that an ultrasound-based imaging method could provide additional information to identify MMD patients with a high risk of stroke [23]. In this research, according to intraoperative MDU assessment, we report that MVV fold increase of RA.ES is a potential risk factor for postoperative CHS. For cases with MVV fold changes larger than 2.7-fold, strict control of blood pressure is necessary, something for which there have been no similar reports published before. Furthermore, MVV fold increase is positively correlated with diabetes and infarction history, but negatively correlated with the onset of symptoms (*p* < 0.05). RA selection among PSCAs that harbor lower MVV might influence the occurrence of postoperative CHS. In other words, the PSCA with the highest MVV has always been selected as the RA in our center. Morcos et al. have revealed that the routine use of intraoperative ultrasonography measurements provides a comprehensive understanding of the hemodynamic changes between different variations of direct bypass techniques, indicating the possibility of one donor artery being used to supply two recipient arteries [24]. Consequently, quantitative analysis of intraoperative ultrasonography has allowed us to evaluate hemodynamics change and to predict the occurrence of postoperative CHS. The accurate control of the MVV fold change of RA.ES corresponding to different strategies demands further research.

## 7. Limitations

This study had some limitations, given as follows. First, this preliminary research is limited by the limited size of MMD of its sample of patients and by the selection bias deriving from reliance on a single-center data source. Second, to assess the hemispheric hemodynamics changes before and after bypass, the pre-infarction stage based on CTP relied upon a semi-quantitative measurement of the hypoperfusion status, with no specific value given as a criterion. As a result, the diagnosis of CHS might not be objectively accurate enough. Third, we speculate that further investigation of the deep layer vascular network of the brain will be meaningful. The MRI technique of territory ASL (T-ASL) might be an option; one which could precisely portray the revascularization territory after bypass. Fourth, the potential value of MVV for the prediction of postoperative hemorrhage is unclear. Thus, a correlation between data deriving from intraoperative video angiography and doppler US will be the cornerstone of our future research.

## 8. Conclusions

Left-operated hemisphere, advanced Suzuki stage, and postoperative fold increase of MVV in RA.ES are potential risk factors for postoperative CHS. Quantitative analysis of intraoperative MDU was useful for evaluating hemodynamics and predicting CHS. This current study offers data and a perspective for choosing MMD candidates and predicting postoperative CHS.

## Figures and Tables

**Table 1:** Demographic data and clinical characteristics.

Characteristic	Values
No. of patients	101
No. of hemispheres	106
Age, years	50.03 ± 8.79
Gender (male)	56 (52.83%)
Onset type	
Hemorrhagic	20 (18.87%)
Ischemic	86 (81.13%)
Surgical side (left)	52 (49.05%)
Previous infarction	61 (57.55%)
Hypertension	52 (49.06%)
Diabetes	18 (16.98%)
Suzuki stage	
I	13 (12.26%)
II	26 (24.53%)
III	39 (36.79%)
IV	16 (15.09%)
V	8 (7.55%)
VI	4 (3.77%)
Puff smoke	
None	19 (17.92%)
Sparse	35 (33.02%)
Dense	52 (49.06%)

Values represent the number of hemispheres (%) unless otherwise indicated. Mean values are presented ± SD.

**Table 2:** Detailed information of the patients with cerebral hyperperfusion.

No	Sex	Age	HT	DB	SS	MV	FD	RA	BG	Cor	Syl	Op Side	Symptoms	Time
1	M	35	0	0	5	sparse	sylvian	7	50	23	83	lt	aphasia	Po3
2	F	57	1	0	6	sparse	Cortex	8	40	20	23	lt	aphasia	
3	M	49	0	1	3	dense	Cortex	3	77	28	73	lt	Seizures	Po5
4	M	52	1	0	3	dense	Cortex	5	70	8	27	lt	verbal dys, hemorrhage	Po2
5	F	39	1	0	6	none	sylvian	7	83	32	30	rt	headache, verbal dys	Po3
6	M	49	1	1	3	dense	sylvian	7	73	53	50	rt	hemorrhage	Po2
7	F	35	1	1	2	sparse	Cortex	1	63	70	70	rt	verbal dys	Po1
8	F	52	1	0	3	dense	Cortex	8	47	1	50	lt	aphasia	Po2
9	M	35	0	0	3	dense	sylvian	9	24	24	17	rt	headache	Po2
10	F	60	0	0	4	dense	Cortex	4	49	15	30	lt	verbal dys	Po3
11	M	40	0	1	4	dense	sylvian	3	67	53	80	rt	headache	Po2
12	M	60	0	0	4	dense	Cortex	4	54	5	13	lt	headache	Po2
13	F	51	0	0	2	sparse	sylvian	5	26	10	15	lt	verbal dys	Po4
14	F	71	1	0	5	sparse	Cortex	7	47	3	21	lt	aphasia	Po5
15	F	46	0	0	5	sparse	sylvian	8	57	6	75	lt	verbal dys	Po3
16	M	56	0	0	3	dense	Cortex	2	27	2	17	lt	SAH, aphasia	Po15 minPo3

HT = hypertension, DB = diabetes, SS = Suzuki stage, MV = moyamoya vessel, FD = flow direction, RA = recipient artery, BG = bypass graft, Cor = cortex, Syl = sylvian, Op = operation, lt = left, rt = right, dys = dysfunction, Po = postoperative.

**Table 3:** Logistic regression analysis of potential predictors for postoperative CHS after direct bypass.

	CHS		*p* Value		
Variables	No (*n* = 90)	Yes (*n* = 16)	Univariate	Multivariate	OR (95%CI)
Gender (male, *n*, %)	49 (54.4%)	7 (43.8%)	0.588 ^a^		
Age (years)	50.79 ± 9.13	49.19 ± 10.45	0.529 ^b^		
Op side (left, *n*, %)	41 (45.6%)	11 (68.8%)	0.108 ^a^	0.043 *	4.58 (1.05–19.97)
Onset type			0.990 ^a^		
Hemorrhagic	17 (18.9%)	3 (18.8%)			
Ischemic	73 (81.1%)	13 (81.3%)			
Post infarction	49 (54.4%)	12 (75.0%)	0.172 ^a^		
Hypertension	45 (50.0%)	7 (43.8%)	0.788 ^a^		
Diabetes	14 (15.6%)	4 (25.0%)	0.467 ^a^		
Suzuki stage			0.008 ^c^*	0.017 *	5.47 (1.99–15.05)
Early	37 (41.1%)	2 (12.5%)	0.046	—	ref
Middle	46 (51.1%)	9 (56.3%)	0.705	0.028 *	13.40 (1.32–136.62)
Advanced	7 (7.8%)	5 (31.3%)	0.017 *	0.005 *	37.20 (3.07–451.30)
Puff smoke			0.281 ^c^		
None	18 (20.0%)	1 (6.3%)			
Sparse	29 (32.2%)	6 (37.5%)			
Dense	43 (47.8%)	9 (56.3%)			
PSCAs direction			0.766 ^a^		
Sylvian	43 (47.8%)	7 (43.8%)			
Cortex	47 (52.2%)	9 (56.3%)			
Pre MVV (RA)	11.24 ± 6.42	7.12 ± 4.95	0.007 ^b^*	0.064	0.88 (0.78–1.33)
Post MVV (RA)					
RA.ES	36.23 ± 23.81	41.56 ± 26.68	0.421 ^b^	0.186	
Post/pre fold	2.1 (1.40, 6.25)	4.75 (2.30, 11.10)	0.008 ^c^*	0.003 *	1.17 (1.06–1.30)
RA.LS	22.51 ± 16.06	22.06 ± 20.83	0.922 ^b^	0.278	
Post/pre fold	2.0 (1.17, 3.23)	2.25 (0.83, 6.67)	0.694 ^c^	0.651	
Post MVV (bypass)	51.41 ± 28.61	53.37 ± 18.23	0.722 ^b^	0.541	

Values represent number of hemispheres (%) unless otherwise indicated. Mean values are presented ± SD. Op = operative, Ref = reference, MVV = mean velocity value, Pre = preoperative, Post = postoperative, PSCAs = parasylvian cortical arteries, “a” represents Chi-square test, “b” represents independent *t* test, “c” represents rank test. “*” *p* < 0.05.

**Table 4:** Correlation analysis of RA.ES hemodynamics and other variables.

RA.ES	Gender	Age	Onset Type	Pre Infarction	HT	DB	MV	SS
r	−0.075	−0.122	−0.222 *	0.252 **	0.085	0.308 **	−0.075	−0.143
*p*	0.442	0.213	0.022	0.009	0.386	0.001	0.442	0.144

HT = hypertension, DB = diabetes, MV = moyamoya vessel, SS = Suzuki stage, RA.ES = recipient artery entering sylvian. “*” *p* < 0.05, “**” *p* < 0.01. Male, with ischemic onset, no hypertension, no diabetes, no moyamoya vessels, and early Suzuki stage was set as reference.

## Data Availability

The datasets generated and/or analyzed during the current study are available from the first author on reasonable request (Zhiyong Shi, szy1195156829@aliyun.com).

## Abbreviations

MMD = moyamoya disease, ICA = internal carotid artery, EC–IC = extracranial–intracranial, STA–MCA= superficial temporal artery–middle cerebral artery, TNDs = transient neurological dysfunction, CHS = cerebral hyperperfusion syndrome, PSCAs = para-sylvian cortical arteries, RA = recipient artery, MDU = microvascular doppler ultrasonography, ICG = indocyanine green, CTP = CT perfusion, MRA = MRI angiography, rCBV = regional cerebral blood volume, rCBF = regional cerebral blood flow, MTT = mean transit time, TTP = time to peak, MVV = mean velocity value, RA.ES = recipient artery entering sylvian, RA.LS = recipient artery leaving sylvian, DWI = diffusion-weighted imaging, OR = ratio of Odds (OR), CI = confidence interval, ASL = arterial spin labeling, T-ASL = territory ASL.
